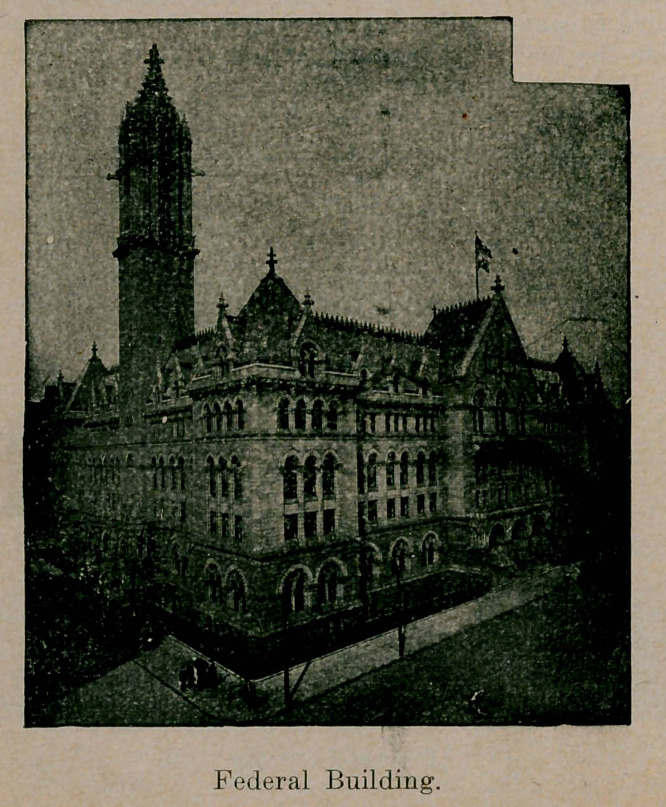# Our Contemporaries

**Published:** 1915-04

**Authors:** 


### OUR CONTEMPORARIES.

The International Hospital Record, published in Detroit, at varying intervals for the last 18 years, has been merged with Modern Hospital of St. Louis and Chicago.

The May issue of the Medical Review of Reviews will be a woman's number, all original articles being by distinguished women physicians. The Buffalo Medical Journal published such an issue several years ago, even the editorial and news departments being assigned to a temporary editorial staff of women. Will the women of western New York co-operate with us in preparing another similar issue?

## Figures and Tables

**Figure f1:**